# The Dyadic Effects of Self-Efficacy on Quality of Life in Advanced Cancer Patient and Family Caregiver Dyads: The Mediating Role of Benefit Finding, Anxiety, and Depression

**DOI:** 10.1155/2022/3073358

**Published:** 2022-09-13

**Authors:** Qian Cao, Jiali Gong, Meizhen Chen, Yi Lin, Qiuping Li

**Affiliations:** ^1^Wuxi School of Medicine, Jiangnan University, Wuxi, Jiangsu, China; ^2^Affiliated Hospital, Jiangnan University, Wuxi, Jiangsu, China

## Abstract

**Objectives:**

To explore the dyadic interdependence of self-efficacy, benefit finding, anxiety, depression, and QOL in cancer patient (CP) and family caregiver (FC) dyads, and to ascertain the dyadic effects of self-efficacy on quality of life (QOL) in CP-FC dyads.

**Methods:**

Conducted from November 2014 to December 2015, participants comprised 772 CP-FC dyads. The study surveyed participant characteristics, self-efficacy, benefit finding, anxiety, depression, and QOL. Data were analyzed using Pearson's correlation, *T*-test, and actor-partner interdependence mediation model (APIMeM).

**Results:**

CPs' self-efficacy was positively correlated with both their own and FCs' benefit finding and mental component summary (MCS), and negatively associated with anxiety and depression (all Ps < 0.01, |*r*| = 0.144∼0.432). However, CPs' self-efficacy was only positively correlated with their own physical component summary (PCS) (*r* = 0.193), but not FCs' PCS. The same profile was identified in FCs' self-efficacy (all Ps < 0.01, |*r*| = 0.100∼0.468). FCs reported higher levels of self-efficacy and PCS compared to CPs (both Ps < 0.001). Significant positive correlations (*r* = 0.168–0.437) were identified among all paired variables in CP-FC dyads (all Ps < 0.001). To some extent, dyads' self-efficacy influences dyads' MCS and PCS through improving positive emotions (benefit finding) and relieving negative emotions (anxiety and depression).

**Conclusions:**

Study findings not only support the dyadic interdependence of self-efficacy, benefit finding, anxiety, depression, and QOL in CP-FC dyads but confirm the hypothesis that dyads' self-efficacy may impact their MCS/PCS via an indirect approach to improve benefit finding and relieve anxiety and/or depression in CP-FC dyads.

## 1. Introduction

A cancer diagnosis, particularly a diagnosis of advanced cancer, cancer treatment, and survivorship are adverse and stressful events for both cancer patients (CPs) and their family caregivers (FCs) [1]. Advanced cancer generally denotes incurable cancer, as the CP's situation gradually deteriorates [1]. During the stressful advanced cancer trajectory, both CPs and their FCs need to cope together and adjust to these adversities of profound physical, emotional, and social adversity, including psychological distress, fatigue, and impaired quality of life (QOL), imposed by advanced cancer and/or caregiving [2–5].

Fortunately, as they progress and adapt to the coping process, CP-FC dyads may thrive and undergo positive experiences, such as improved self-efficacy, adjusted life priorities, and increased family interactions [6–9]. This phenomenon is mirrored in the revised stress and coping model (SCM), in that positive emotions/effects co-occur along with the inevitable negative emotions/effects inherent in the circumstance of an extremely stressful event, e.g., advanced cancer and caregiving [10]. As an appraisal-based model, SCM [11] proposes that individuals' cognitive appraisals of their stress (primary appraisal) and coping (secondary appraisal) experiences trigger the coping process. In the context of advanced cancer coping process, primary appraisals refer to individuals, CPs and FCs in this case, who evaluate the personal significance of the stressful event like advanced cancer [1], e.g., the ways in which cancer or caregiving trajectory affects or threatens their lives and livelihood. Secondary appraisals are evaluations of their capability to manage cancer or caregiving consequences, e.g., their self-efficacy in coping with cancer or the caregiving situation [1]. Self-efficacy in coping with cancer is conceptualized as a specific construct that facilitates CPs' ability to adjust their behaviors, and stabilize their emotions in the trajectory of coping with cancer, ranging from cancer diagnosis and cancer treatment to survivorship care [12–15].

Indeed, evidence has shown that self-efficacy plays an important role in CPs' response to stressors associated with a cancer diagnosis, treatment, and survivorship [1, 15]. Findings in the area of psychosocial oncology have also revealed the fact that the higher the levels of self-efficacy, the fewer the negative emotions, e.g., anxiety and depression, and the better the QOL [13, 15–18]. It deserves noting that the majority of these studies targeted CPs or cancer survivors. However, few researchers have discovered the relationship between self-efficacy and positive emotions, e.g., benefit findings [19]. Study gaps also exist concerning the possible mechanism contributing to the interrelationships between these variables, e.g., whether emotional outcomes (e.g., positive or negative emotions) can mediate the effects of self-efficacy on favorable outcomes, e.g., improved QOL remains unknown.

Considering interpersonal influences on cancer-related outcomes in CP-FC dyads, the Actor-Partner interdependence model (APIM) allows researchers to explore the impact of an individual's predictor variable (e.g., CP self-efficacy) on his/her own outcome variables (e.g., QOL of CPs, named the actor effect) and on the partner's outcome variables (e.g., FC QOL, the partner effect) using dyadic data [20, 21]. Further, an extension of APIM, the Actor-Partner Interdependence Mediation Model (APIMeM), makes it possible to estimate the mediation or indirect effects of the dyadic impact by adding a third variable [22], e.g., to explore the possible mediating effects of positive or negative emotions on the important impact of CP-FC dyads' self-efficacy on their QOL in the current study. Indeed, APIM or APIMeM has been profoundly applied in a dyadic context of CP-FC dyads, showing dyadic interdependence in various variables, indicating psychosocial well-being, e.g., depression, anxiety, positive affect, and personal growth [2–4, 8, 23–25] Nevertheless, no study to date has reported the interdependence between the variables of self-efficacy, benefit finding, anxiety, depression, and QOL in Chinese CP-FC dyads using APIM or APIMeM analysis.

To fill in these study gaps, the current study was intended to investigate the interdependences of these outcome variables, e.g., self-efficacy benefit finding, anxiety, depression, and QOL, in CP-FC dyads from a dyadic perspective. Accordingly, the study aims were: (i) to assess correlations between dyads' (both CP and FC) self-efficacy and other variables, e.g., benefit finding, anxiety, depression, and QOL, (ii) to examine the mutual relationship of the outcome variables, including self-efficacy, benefit finding, anxiety, depression, and QOL, between CP-FC dyads, and (iii) to ascertain the effects of self-efficacy on QOL in CP-FC dyads. It was hypothesized that dyads' self-efficacy would be positively associated with dyads' benefit finding and QOL, negatively correlated with dyads' anxiety and depression; dyadic interdependence of self-efficacy, benefit finding, anxiety, depression, and QOL would exist in CP-FC dyads; and self-efficacy could influence dyads' QOL via improving positive emotions (benefit finding) and relieving negative emotions (psychological distress).

## 2. Methods

The data in this analysis were extracted from a project study on Chinese CP-FC dyads. While the previous study explored the factor structure of the 17-item Beneﬁt Finding Scale (BFS) [26], the Cancer Behavior Inventory-brief (CBI–B) [27], and identified moderating factors for the beneﬁt finding associations in CP- FC dyads [6], the main focus of this analysis was to disclose the dyadic impact of self-efficacy on QOL in CP-FC dyads. Participants included CP-FC dyads. CPs had a confirmed cancer diagnosis and were being cared for by a family caregiver, namely FC. Moreover, both CPs and FCs were adults (>18 years old).

Regarding the sample size, the 772 CP-FC dyads that were included were calculated based on the sample requirement for factor analysis in the previous study [26]. In addition, according to the APIM power analysis, there is virtually 1.00 power to detect an actor and/or partner effect for Person 1 or Person 2 of a size 0.150 in a standardized regression coefficient [28].

### 2.1. Instruments

A self-developed inquiry form was used to solicit CP-FC dyad characteristics, e.g., age, gender, FC relationship with CP, employment status, cancer type, and average time since diagnosis (Table 1). Additionally, the following four types of variables were collected:

- *Self-efficacy*: Self-efficacy was assessed by the 12-item CBI-B [15]. CBI-B is designed to estimate participants' confidence in their ability to perform a particular activity associated with self-efficacy in the cancer-coping process [15]. Items were calculated using a nine-point option (1–9), indicating different confidence levels ranging from not at all (1), moderately (5), to totally (9). The 12-item scores were summed to provide the CBI-B total score, with higher scores (ranging between 12 and 108) signifying better self-efficacy. A previous study on the Chinese version CBI-B (CBI–B/C) has offered a satisfactory single-factor construct and good internal stability in CP-FC dyads [27].
- *Benefit finding*: The 17-item BFS was used to examine benefit findings [29]. The BFS has been widely applied and validated in either CPs [29, 30] or FCs [31]. The BFS items are scored using a five-point (1–5) scale option, and the BFS total score is reached by adding up the 17 individual items that were included. The higher the BFS score (range 17–85), the better the benefit finding. A previous study on the Chinese version of BFS (C–BFS) has established a three-factor validity and good internal stability in CP-FC dyads [26].
- *Anxiety and depression*: The Hospital Anxiety and Depression Scale (HADS) was applied to evaluate anxiety and depression [32]. A four-point (0–3) scale option was used to score each item. Anxiety and depression scores were generated by adding up the seven individual items that were included, with higher scores demonstrating higher levels of anxiety or depression. Study findings using the HADS Chinese version in Chinese CP-FC dyads have confirmed its applicability and validity [33].
- *QOL*: QOL was calculated using the medical outcomes study 12-item short-form version 2 (SF-12 v2) [34]. According to the SF-12 score guidebook [35], the two aggregate scores of mental component summary (MCS) and physical component Summary (PCS) were reached, with a scale ranging between 0 and 100. The higher the score, the better the QOL [35].

### 2.2. Procedures

After obtaining ethical authorization from the university's ethics board (HSEARS201410005), and written informed consent from eligible CP-FC dyads, the CP-FC dyads were then guided to independently fill out the survey. The survey was administered from November 2014 to December 2015 at two hospitals in Wuxi, China.

### 2.3. Data Analysis

SPSS and Amos version 22.0 were applied to analyze the data. CP-FC dyad characteristics and the four outcome measures (CBI–B, BFS, HADS, and SF-12) are presented using descriptive statistics. The mean differences in paired outcome measures between CPs and FCs were examined using paired *T*-test. Pearson correlations were used to investigate variable correlations between CPs and FCs.

The APIMeM was applied to uncover the dyadic effects of self-efficacy on dyads' QOL [20–22], using self-efficacy to predict QOL through benefit finding (Figure S1) and psychological distress respectively (Figure S2). Given the fact that anxiety and depression are generally highly associated with one another (in the current sample, values of *r* were 0.833 and 0.835 for CPs and FCs respectively), a latent variable, designated by anxiety and depression, was treated as psychological distress. In the APIMeM, 1 and 2 were labels for CPs and FCs, respectively. The effect of self-efficacy on benefit finding (Figure S1)/psychological distress (Figure S2) is labeled “a,” the effect of benefit finding/psychological distress on QOL as “b,” and the effect of self-efficacy on QOL as “c.” The actor effect [the impact of an individual's predictor variable (e.g., self-efficacy of CPs) on his/her own outcome variables (e.g., QOL of CPs)] and partner effect [the impact of an individual's predictor variable (e.g., self-efficacy of CPs) on the partner's outcome variables (e.g., QOL of FCs)] were indexed as A and P, respectively [20–22]. The significance of total effect or total indirect effect (IEs) was evaluated by conducting the bias-corrected bootstrap test with a 95% confidence interval.

## 3. Results

Among the 772 dyads, all CPs had advanced cancer. And more than 90% of participants were married. The majority of FCs (61.4%) were spouses. Approximately three-fifths (57.0% and 60.5% for CPs and FCs, respectively) were employed (Table 1).

### 3.1. Correlations between Dyads' Self-Efficacy and Other Variables

Table 2 shows correlations between dyads' (both CP and FC) self-efficacy and other outcome variables in CPs and FCs. At the individual level, CP findings showed that self-efficacy was positively related to benefit finding, MCS, and PCS (*r* = 0.193∼0.432), and negatively associated with anxiety (*r* = −0.418) and depression (*r* = −0.430). The same profile was identified for FC variables (|*r*| = 0.241∼0.468).

At the dyadic level, correlations between dyads' self-efficacy and other variables in their partners showed that self-efficacy in CPs was significantly correlated with other FC variables (CP1: Self-efficacy column), including benefit finding (*r* = 0.194), anxiety (*r* = −0.187), depression (*r* = −0.188), and MCS (*r* = 0.144), with the exception of borderline correlation with PCS (*r* = 0.071, *P*=0.058). The same profile was identified for self-efficacy in FCs (FC1: Self-efficacy column), where self-efficacy in FCs was significantly correlated with CPs' benefit finding (*r* = 0.319), anxiety (*r* = −0.164), depression (*r* = −0.190), and MCS (*r* = 0.100), but not PCS. All of the above significant correlations are at the *P* < 0.001 level, with one exception of *P*=0.007 for a correlation between FCs' self-efficacy and CPs' MCS.

### 3.2. Dyadic Relationship of Outcome Variables

Table 3 presents Pearson correlations and paired differences of all of the outcome variables between CPs and FCs. Significant positive correlations (*r* = 0.168–0.437) were identified among all of the paired variables, including self-efficacy, benefit finding, anxiety, depression, MCS, and PCS, in CP-FC dyads (all Ps < 0.001). In terms of differences, FCs reported higher levels of self-efficacy and PCS compared to CPs (both Ps < 0.001).

### 3.3. Dyadic Impact of Self-Efficacy on QOL

Part (a) in Table 4 and Figure S3 show the effects of self-efficacy on QOL mediated by benefit findings. For the effects of self-efficacy on benefit finding, there were four significant positive actor effects (a A1, a A2, all Ps < 0.001), and two significant positive CP partner effects (a P1, all Ps < 0.001). For the b effects, there was one significant positive FC actor effect (b A2, *P*=0.024), and one borderline negative CP partner effect (bP1, *P*=0.072) from benefit finding to MCS in submodel MCS. In submodel PCS, one borderline positive FC partner effect (bP2, *P*=0.066) from benefit finding to PCS was identified. For the c' effects of self-efficacy on QOL: four significant positive direct actor effects of self-efficacy on both MCS and PCS (c'A1, c'A2, all Ps < 0.001) were identified.

Part (b) in Table 4 and Figure S4 present the effects of self-efficacy on QOL, mediated by psychological distress. For the effects of self-efficacy on psychological distress: four significant negative actor effects (a A1 and a A2, all Ps < 0.001) and two negative FC partner effects (a P2, all Ps < 0.05) were identified. For the b effects from psychological distress to QOL: all four negative actor effects (b A1 and b A2, all Ps < 0.001) were significant. In submodel MCS, one borderline negative CP partner effect (bP1, *P*=0.069) was recognized. For the c' effects of self-efficacy on QOL, two significant positive actor effects (c'A1 and c'A2, *P*=0.004 and 0.001 respectively) in submodel MCS, and one significant positive FC actor effect (c'A2, *P* < 0.001) in submodel PCS were found.

Table 5 presents the bias-corrected bootstrap tests of the total effects, total IEs, and direct effects. In model a: effects of self-efficacy on QOL mediated by benefit finding are shown in 5(a) in Table 5. For actor effects: there were four significant CP actor effects (total and direct effects, all Ps = 0.001), four significant FC actor effects (total and direct effects, all Ps = 0.001), and one borderline FC total IEs (*P*=0.051) in submodel MCS. For partner effects: one borderline CP total IEs (*P*=0.066) in submodel MCS, and one borderline FC total IEs (*P*=0.056) in submodel PCS were identified.

In model b: effects of self-efficacy on QOL mediated by psychological distress are presented in part (b) in Table 5. For actor effects: apart from CP direct effect c', all other 11 actor effects (11/12) in both submodels were statistically positive significant (*P*=0.011 ~ 0.001). For partner effects: one significant positive FC total IEs (*P*=0.012) in submodel MCS, and two borderline FC partner effects (positive total IEs, *P*=0.078, and negative direct effect c', *P*=0.079) in submodel PCS were identified.

Part (c) in Table 5 shows the total IEs of self-efficacy on anxiety or depression. For actor effects: all eight IEs in both CPs and FCs were negatively significant (all Ps = 0.001). For partner effects: two negative significant FC total IEs (all Ps < 0.05) in submodel MCS, and two negative borderline FC total IEs (*P*=0.068, and 0.071 respectively) in submodel PCS were identified. It is worth noting that although no significant CP partner effects were identified, all four CP partner effects were positive in terms of their effect direction, which are different from other effects, e.g., CP and FC actor effect, and FC partner effect.

## 4. Discussion

This study aimed to scrutinize the relationships between the variables of self-efficacy, benefit finding, anxiety, depression, and QOL, and to ascertain the dyadic influence of self-efficacy on QOL in CP-FC dyads. Study findings, generally, support our hypothesis that dyads' self-efficacy is positively associated with dyads' benefit findings and QOL, and negatively correlated with dyads' anxiety and depression. They also support that dyadic interdependence exists between variables of self-efficacy, benefit finding, anxiety, depression, and QOL; and that self-efficacy could exert an impact on dyads' QOL through improving positive emotions (benefit finding) and relieving negative emotions (psychological distress). The following discussion will mainly focus on the three corresponding aspects, as indicated in the study aims and hypothesis.

### 4.1. Correlations between Dyads' Self-Efficacy and Other Variables

At the individual level of CPs or FCs, findings of significant positive associations between self-efficacy and their own QOL, and negative associations between self-efficacy and anxiety/depression, were consistent with other study findings in various cancer populations, as described earlier [13, 15–18]. Few studies have focused on the associations between self-efficacy and benefit finding in either CPs or FCs. In general, the significant dyadic correlations between dyads' self-efficacy and other variables are in line with the findings of other studies on couples dealing with cancer together [36, 37]. Another study revealed that self-efficacy is a factor that moderates benefit-finding correlations in CP-FC dyads [6]. These findings may indicate that dyadic interdependence exists in CP-FC dyads in their journey of dealing with cancer as a unit.

### 4.2. Dyadic Relationship of Outcome Variables

Findings of the significant correlations between all of the paired variables, e.g., self-efficacy, benefit finding, anxiety, depression, and QOL, between CP-FC dyads are similar to previous findings in a sample of cancer couple dyads [36]. These findings further confirm the dyadic interdependence between CPs and FCs in terms of self-efficacy, benefit finding, anxiety, depression, and QOL.

In terms of differences in the paired outcome variables between CPs and FCs, it is reasonable that CPs experienced lower levels of self-efficacy and PCS than FCs because CPs suffer from the effects of advanced cancer and its treatment. Findings of lower levels of PCS in CPs than in FCs are in line with another sample finding in Chinese couples coping with advanced cancer [4]. This generally poor QOL in CP-FC dyads serves as a call for further studies, to develop more effective interventions for improving CP-FC dyad QOL.

### 4.3. Dyadic Impact of Self-Efficacy on QOL

Discoveries of the two APIMeM models further support the interdependence of self-efficacy, benefit finding, anxiety, depression, and QOL in CP-FC dyads. This indicates that dyads' self-efficacy may impact their QOL via an indirect approach to improving positive emotions (benefit finding) and relieving negative emotions (psychological distress) in CP-FC dyads. The significant or borderline total IEs findings using the bias-corrected bootstrap method further support the mediation effects of positive (benefit finding) and negative (psychological distress) emotions on the impact of dyads' self-efficacy on their QOL.

In both model a and model b, the overall significant positive c' actor effects (seven out of eight in two models) of self-efficacy on QOL, correspond relatively well to another study, in which each person's (advanced CPs and their FCs) self-efficacy positively influenced their own mental and physical health [14]. However, the overall negative c' partner effects (seven out of eight in two models) of self-efficacy on QOL, although not statistically significant, were unanticipated.

Further analysis of model a (part (a) in Table 4) revealed a positive influence of self-efficacy on benefit finding (A1, A2, and P1), with the latter (benefit finding) again positively influencing QOL (b A1 and b A2). These findings are both explicable and meaningful. One possible explanation may because benefit finding is by far the most commonly reported kind of meaning-focused coping [10]. On the contrary, the results of the negative partner effects of self-efficacy on benefit finding (a P2), and benefit finding on QOL (bP1 and bP2) were unexpected. The reason is that all of the above unexpected negative partner effects, including a P2, bP1, bP2, c'P1, and c'P2, may partially lie in the interdependence of active coping between CPs and FCs [9]. The scenario may be as follows: overdone active coping in one partner, e.g., FC caregiving, may lead to less active coping in the other, e.g., the CP's own coping. Therefore, this contradiction in the interdependence between CP and FC dyads may contribute to the generally negative impact of FC coping (self-efficacy and/or benefit finding) on CP's QOL and/or benefit finding, and vice versa. The evidence of a positive direction in the CP partner effect (a P1) of FC self-efficacy on CP psychological distress in model b (part (b) in Table 4) may also support the above perspective on the interdependence of active coping in CP-FC dyads. Nevertheless, this is a reminder that further in-depth exploration, to advance a profound understanding of the complex dyadic interdependence in terms of self-efficacy, benefit finding, anxiety, depression, and QOL in CP-FC dyads, and particularly, how to improve the positive interdependence of dyadic active coping by CP-FC dyads, is required.

In addition, due to the positive coping property of self-efficacy in stressful events, discoveries of the negative total IEs of self-efficacy on anxiety and depression (part (c) in Table 5), e.g., CP actor effects, and FC actor/partner effects, are rational. However, the positive CP partner effects, although not statistically significant, are again surprising. This may partly result from the above-described mechanism in dyadic interdependence of active coping by CP-FC dyads. Nevertheless, these findings may further support the study hypothesis, that self-efficacy may influence QOL by way of an indirect tactic to relieve negative emotions (psychological distress) in CP-FC dyads.

### 4.4. Limitations

First, the cross-sectional survey study restricts the probability of providing outcome variables' progress trajectory. Second, participants' unique cultural background, e.g., Chinese CP-FC dyads, limits the generalization of the study findings to other populations with diverse cultural backgrounds. Future longitudinal studies targeting participants in dissimilar cultures are required.

### 4.5. Implications for Practice

Notwithstanding the aforementioned limitations, the findings may point to the following prospective implications for practice. The dyadic interdependence of related variables in CP-FC dyads highlights the importance, for healthcare professionals in cancer practice, of treating CP-FC dyads as a coping unit. The APIMeM findings on the influences of self-efficacy on QOL further advance the significant recognition of the importance of refining dyadic self-efficacy in future intervention studies aimed at improving CP-FC dyad QOL. In caring for CP-FC dyads, dyadic-based interventions, comprised of such elements as refining dyads' self-efficacy and cultivating positive dyadic active coping interrelationships, are highly recommended for improving dyads' QOL.

## 5. Conclusion

The study findings support dyadic interdependence among variables of self-efficacy, benefit finding, anxiety, depression, and QOL in CP-FC dyads. In addition, the findings also support the hypothesis that CP-FC dyads' self-efficacy may influence their QOL via an indirect tactic, by improving positive emotions and relieving negative emotions within CP-FC dyads.

## Figures and Tables

**Table 1:** Characteristics of cancer patients and family caregivers.

Characteristics	Patients [ *n* (%)]	FC [ *n* (%)]

Age (mean ± SD), years	55.1 ± 12.7 (range: 18–88)	48.3 ± 13.4 (range: 18–80)

*Gender*		
Male	403 (52.2)	360 (46.6)
Female	367 (47.5)	411 (53.2)
Missing data	2 (0.3)	1 (0.2)

*Marital status*		
Married	717 (92.9)	702 (90.9)
Divorced	10 (1.3)	2 (0.3)
Widowed	21 (2.7)	2 (0.3)
Never married	24 (3.1)	65 (8.4)
Missing data	0 (0.0)	1 (0.1)

*FC relationship with patients*		
Spouse	474 (61.4)	
Offspring	215 (27.8)	
Parent	20 (2.6)	
Sibling	41 (5.3)	
Other	21 (2.7)	
Missing data	1 (0.2)	

*Education levels*		
Primary school or less	420 (54.4)	323 (41.8)
High school	247 (32.0)	271 (35.1)
University or above	103 (13.3)	174 (22.5)
Missing data	2 (0.3)	4 (0.6)

*Employment status*		
Employed	440 (57.0)	467 (60.5)
Not-employed	327 (42.4)	302 (39.1)
Missing data	5 (0.6)	3 (0.4)

*Type of cancer* ^*†*^		
Breast cancer	79 (10.2)	
Ovarian and cervical cancer	95 (12.3)	
Esophageal and gastric cancer	186 (24.1)	
Colorectal cancer	113 (14.6)	
Liver cancer	69 (8.9)	
Lung cancer	122 (15.8)	
Others	91 (11.8)	
Missing data	17 (2.2)	

Average time since diagnosis (mean ± SD), months	12.9 ± 12.5 (range: 3–192)	

Note: FC = family caregivers; SD = standard deviation. ^†^All cancer patients were diagnosed at an advanced stage of the disease.

**Table 2:** Correlations between self-efficacy and other outcome variables in cancer patients and family caregivers dyads (*n* = 772).

Variables	CP1: self-efficacy	*P* value	FC1: self-efficacy	*P* value
*Variables of cancer patients*				
CP1: self-efficacy	—		0.418	<0.001
CP2: benefit finding	0.432	<0.001	0.319	<0.001
CP3: anxiety	−0.418	<0.001	−0.164	<0.001
CP4: depression	−0.430	<0.001	−0.190	<0.001
CP5: MCS	0.339	<0.001	0.100	0.007
CP6: PCSDSB	0.193	<0.001	0.047	0.215

*Variables of family caregivers*				
FC1: self-efficacy	0.418	<0.001	—	
FC2: benefit finding	0.194	<0.001	0.468	<0.001
FC3: anxiety	−0.187	<0.001	−0.296	<0.001
FC4: depression	−0.188	<0.001	−0.351	<0.001
FC5: MCS	0.144	<0.001	0.281	<0.001
FC6: PCS	0.071	0.058	0.241	<0.001

Note: CP = cancer patients; FC = family caregivers; MCS = mental component summary; PCS = physical component summary.

**Table 3:** Pearson correlations and paired differences of all the outcome variables between cancer patients and family caregivers (*n* = 772).

Outcome variables	Cancer patients (mean ± SD)	Family caregivers (mean ± SD)	*r*	*P* value	*t*	*P* value
Self-efficacy	76.1 ± 19.5	81.2 ± 17.5	0.418	<0.001	−7.473	<0.001
Benefit finding	59.7 ± 13.7	60.1 ± 13.8	0.437	<0.001	−0.788	0.431
Anxiety	8.6 ± 4.5	8.8 ± 4.4	0.338	<0.001	−0.798	0.425
Depression	8.3 ± 4.9	8.5 ± 4.7	0.330	<0.001	−0.879	0.380
MCS	41.3 ± 8.4	41.9 ± 7.9	0.217	<0.001	−1.398	0.163
PCS	36.4 ± 8.9	45.1 ± 8.7	0.168	<0.001	−20.235	<0.001

Note: MCS = mental component summary; PCS = physical component summary; SD = standard deviation.

**Table 4:** Effect estimates for cancer patients and family caregivers.

Effect	Submodel MCS				Submodel PCS			
	Estimate	SE	*P* value	Standard estimate	Estimate	SE	*P* value	Standard estimate
*4(a): effects on QOL mediated by BF (model a)*								
(a): effects (self-E ⟶ BF)								
CP actor effect (a A1)	0.246	0.028	<0.001	0.349	0.246	0.028	<0.001	0.349
FC actor effect (a A2)	0.355	0.031	<0.001	0.451	0.355	0.031	<0.001	0.451
CP partner effect (a P1)	0.122	0.031	<0.001	0.153	0.122	0.031	<0.001	0.153
FC partner effect (a P2)	−0.009	0.027	0.742	−0.013	−0.009	0.027	0.742	−0.013
(b): effects (BF ⟶ QOL)								
CP actor effect (b A1)	0.019	0.026	0.474	0.032	0.014	0.030	0.631	0.022
FC actor effect (b A2)	0.059	0.026	0.024	0.102	0.005	0.029	0.873	0.007
CP partner effect (b P1)	−0.048	0.027	0.072	−0.080	0.008	0.030	0.791	0.012
FC partner effect (b P2)	−0.010	0.026	0.694	−0.018	0.053	0.029	0.066	0.084
(c'): effects (self-E ⟶ QOL)								
CP actor effect (c' A1)	0.144	0.018	<0.001	0.343	0.088	0.021	<0.001	0.194
FC actor effect (c' A2)	0.105	0.021	<0.001	0.230	0.114	0.023	<0.001	0.227
CP partner effect (c' P1)	−0.007	0.021	0.731	−0.015	−0.027	0.024	0.252	−0.054
FC partner effect (c' P2)	0.022	0.018	0.227	0.053	−0.025	0.020	0.209	−0.057

*4(b): effects on QOL mediated by PD (model b)*								
(a): effects (self-E ⟶ PD)								
CP actor effect (a A1)	−0.104	0.009	<0.001	−0.486	−0.101	0.009	<0.001	−0.483
FC actor effect (a A2)	−0.076	0.011	<0.001	−0.308	−0.081	0.011	<0.001	−0.320
CP partner effect (a P1)	0.007	0.010	0.492	0.028	0.006	0.010	0.544	0.025
FC partner effect (a P2)	−0.021	0.009	0.027	−0.095	−0.019	0.010	0.049	−0.085
(b): effects (PD ⟶ QOL)								
CP actor effect (b A1)	−0.921	0.087	<0.001	−0.468	−0.703	0.104	<0.001	−0.324
FC actor effect (b A2)	−0.907	0.078	<0.001	−0.491	−0.636	0.091	<0.001	−0.323
CP partner effect (b P1)	−0.141	0.078	0.069	−0.074	−0.147	0.089	0.101	−0.073
FC partner effect (b P2)	−0.125	0.081	0.123	−0.066	−0.086	0.101	0.398	−0.040
(c'): effects (self-E ⟶ QOL)								
CP actor effect (c' A1)	0.051	0.017	0.004	0.121	0.018	0.021	0.399	0.039
FC actor effect (c' A2)	0.056	0.018	0.001	0.123	0.071	0.021	<0.001	0.141
CP partner effect (c' P1)	−0.027	0.018	0.145	−0.057	−0.031	0.022	0.164	−0.060
FC partner effect (c' P2)	−0.013	0.017	0.428	−0.033	−0.033	0.020	0.105	−0.074

Note: BF = benefit finding; MCS = mental component summary; PCS = physical component summary; PD = psychological distress; QOL = quality of life; SE = standard error; Self-E = self-efficacy; 1 = CP = cancer patients; 2 = FC = family caregivers.

**Table 5:** The bios-corrected bootstrap tests of the total effects, total indirect effects, and direct effects for cancer patients and family caregivers^*∗*^.

Effect	Submodel MCS						Submodel PCS			
	Estimate		95% CI low	95% CI high	*P* value		Estimate	95% CI low	95% CI high	*P* value
*5(a): effects on QOL mediated by BF (model a)*										
CP actor effect										
Total effect	0.149		0.112	0.192	0.001		0.091	0.053	0.133	0.001
Total IEs	0.005		−0.009	0.021	0.452		0.003	−0.012	0.019	0.645
Direct effect c'	0.144		0.104	0.191	0.001		0.088	0.046	0.131	0.001
*FC actor effect*										
Total effect	0.125		0.087	0.157	0.001		0.122	0.079	0.165	0.001
Total IEs	0.020		0.000	0.040	0.051		0.008	−0.012	0.028	0.429
Direct effect c'	0.105		0.065	0.141	0.001		0.114	0.069	0.159	0.001
CP partner effect										
Total effect	−0.022		−0.063	0.017	0.251		−0.023	−0.066	0.025	0.374
Total IEs	−0.015		−0.034	0.001	0.066		0.005	−0.014	0.025	0.646
Direct effect c'	−0.007		−0.051	0.037	0.769		−0.027	−0.074	0.025	0.311
FC partner effect										
Total effect	0.019		−0.013	0.050	0.256		−0.012	−0.048	0.024	0.462
Total IEs	−0.003		−0.017	0.011	0.635		0.013	0.000	0.028	0.056
Direct effect c'	0.022		−0.012	0.056	0.190		−0.025	−0.063	0.014	0.170

*5(b): effects on QOL mediated by PD (model b)*										
CP actor effect										
Total effect		0.149	0.112	0.192		0.001	0.091	0.053	0.133	0.001
Total IEs		0.098	0.072	0.129		0.001	0.073	0.051	0.105	0.001
Direct effect c'		0.051	0.013	0.090		0.011	0.018	−0.029	0.064	0.455
FC actor effect										
Total effect		0.125	0.087	0.157		0.001	0.122	0.079	0.165	0.001
Total IEs		0.069	0.046	0.095		0.001	0.051	0.032	0.075	0.001
Direct effect c'		0.056	0.021	0.089		0.004	0.071	0.029	0.113	0.001
CP partner effect										
Total effect		−0.022	−0.063	0.017		0.251	−0.023	−0.066	0.025	0.374
Total IEs		0.005	−0.020	0.026		0.730	0.008	−0.017	0.030	0.520
Direct effect c'		−0.027	−0.068	0.013		0.184	−0.031	−0.076	0.018	0.221
FC partner effect										
Total effect		0.019	−0.013	0.050		0.256	−0.012	−0.048	0.024	0.462
Total IEs		0.032	0.007	0.057		0.012	0.021	−0.002	0.047	0.078
Direct effect c'		−0.013	−0.048	0.018		0.416	−0.033	−0.070	0.005	0.079

*5(c): total IEs on anxiety and depression (model b)*										
CP actor effect										
Anxiety		−0.104	−0.123	−0.084		0.001	−0.101	−0.121	−0.080	0.001
Depression		−0.112	−0.133	−0.091		0.001	−0.114	−0.135	−0.093	0.001
FC actor effect										
Anxiety		−0.071	−0.090	−0.051		0.001	−0.072	−0.090	−0.053	0.001
Depression		−0.076	−0.099	−0.052		0.001	−0.081	−0.103	−0.056	0.001
CP partner effect										
Anxiety		0.007	−0.014	0.028		0.476	0.006	−0.014	0.026	0.537
Depression		0.007	−0.014	0.029		0.470	0.007	−0.016	0.029	0.543
FC partner effect										
Anxiety		−0.020	−0.037	−0.002		0.036	−0.017	−0.035	0.002	0.068
Depression		−0.021	−0.039	−0.002		0.038	−0.019	−0.038	0.002	0.071

Note: BF = benefit finding; CI = confidence interval; CP = cancer patients; FC = family caregivers; IEs = indirect effects; MCS = mental component summary; PCS = physical component summary; PD = psychological distress. ^*∗*^The bootstrap estimates presented here are based on 2,000 bootstrap samples.

## Data Availability

The authors have full control of all primary data and agree to allow the journal to review the data if requested and are available to readers upon request.

## Supplementary Materials

Supplementary Materials Figure S1: theoretical model in testing the impact of self-efficacy on Quality of life mediated by Benefit finding (Model a); Figure S2: theoretical model in testing the impact of self-efficacy on Quality of life mediated by Psychological distress (Model b); Figure S3: two submodels for testing the impact of self-efficacy on Quality of life mediated by Benefit finding (Model a); Figure S4: two submodels for testing the impact of self-efficacy on Quality of life mediated by Psychological distress (Model b).
