# Supplementary material for: The Dyadic Effects of Self-Efficacy on Quality of Life in Advanced Cancer Patient and Family Caregiver Dyads: The Mediating Role of Benefit Finding, Anxiety, and Depression
Source: J Oncol. 2022 Sep 13;2022:3073358. doi: 10.1155/2022/3073358 (PMC10182880; doi:10.1155/2022/3073358)

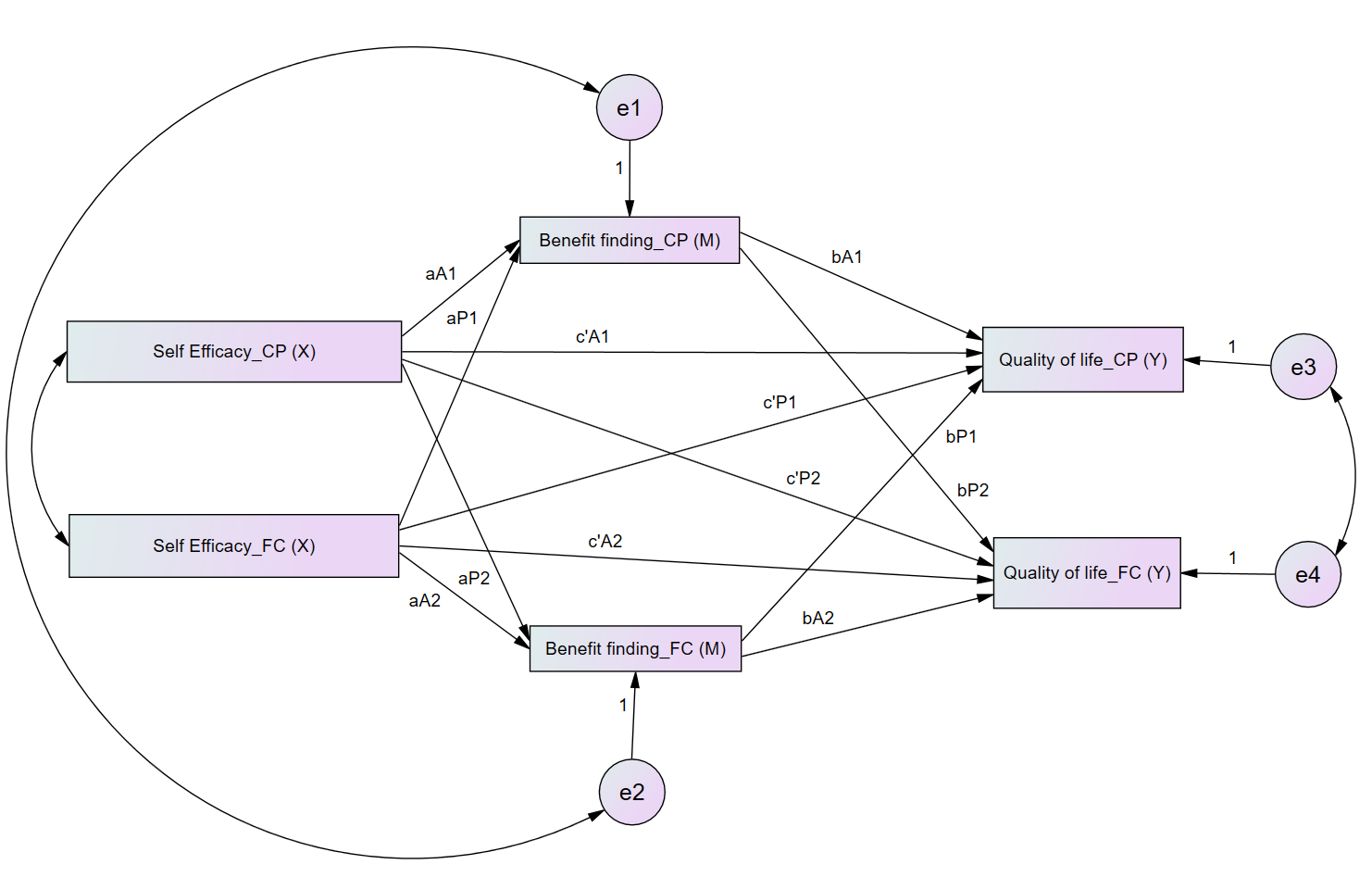


**Figure S1.** Theoretical model in testing the impact of self-efficacy on Quality of life mediated by Benefit finding (Model a).

**Legends:**

Quality of life was replaced by Mental Component Summary (MCS: submodel MCS) and Physical Component Summary (PCS: submodel PCS).

In this mediation model, the effect from self-efficacy to benefit finding is designated as a, the effect from benefit finding on Quality of life as b, and the effect from self-efficacy on quality of life as c’.

A stands for Actor effects; P stands for Partner effects;

1 stands for Cancer patients; 2 stands for family caregivers

CP= Cancer Patients, FC=Family Caregivers


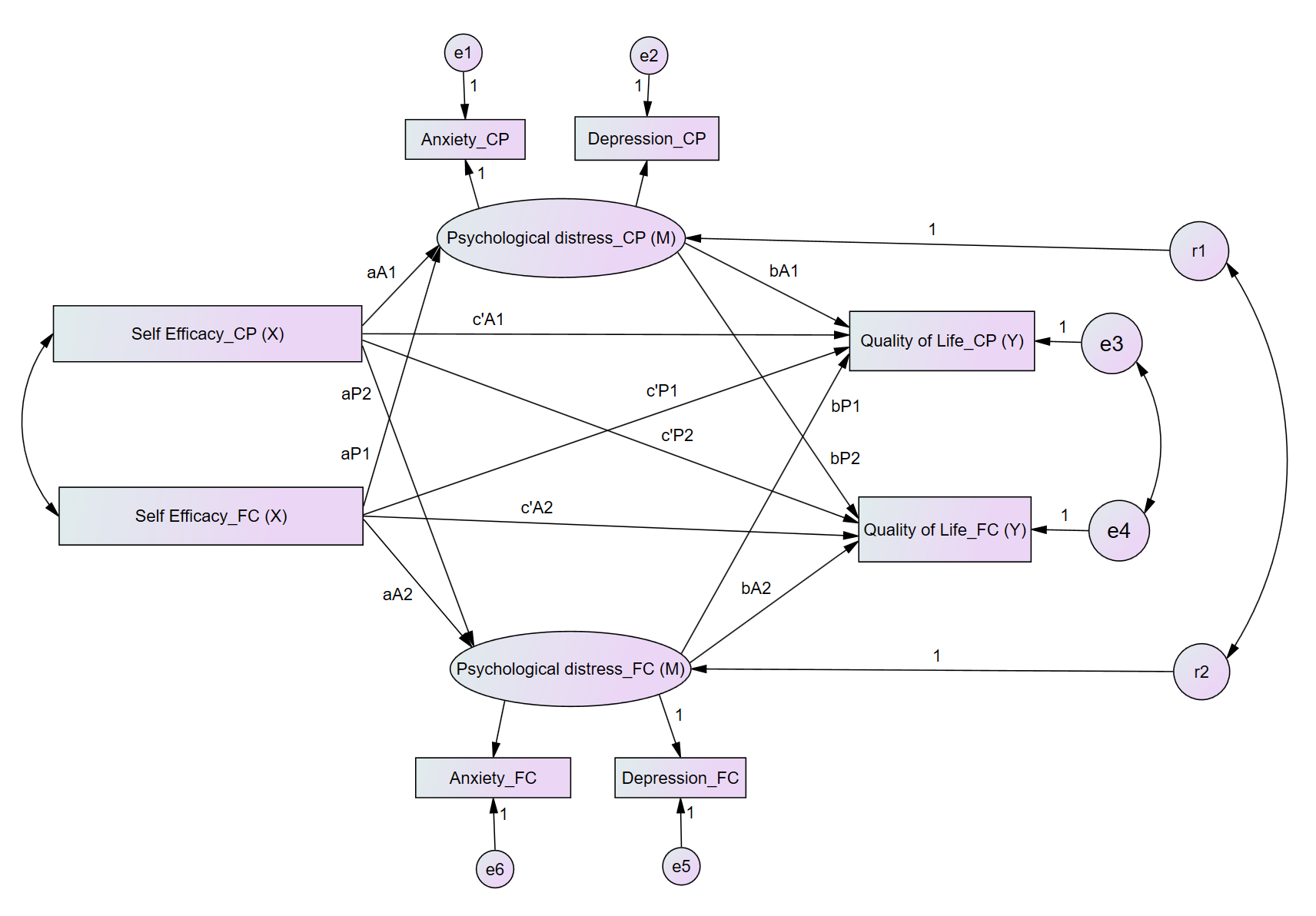


**Figure S2.** Theoretical model in testing the impact of self-efficacy on Quality of life mediated by Psychological distress (Model b).

**Legends:**

Psychological distress was treated as a latent variable indicated by anxiety and depression.

Quality of life was replaced by Mental Component Summary (MCS: submodel MCS) and Physical Component Summary (PCS: submodel PCS).

In this mediation model, the effect from self-efficacy to psychological distress is designated as a, the effect from psychological distress on Quality of life as b, and the effect from self-efficacy on quality of life as c’.

A stands for Actor effects; P stands for Partner effects;

1 stands for Cancer patients; 2 stands for family caregivers

CP= Cancer Patients, FC=Family Caregivers

**Figure S3.** Two submodels for testing the impact of self-efficacy on Quality of life mediated by Benefit finding (Model a)

*Model a: submodel MCS*


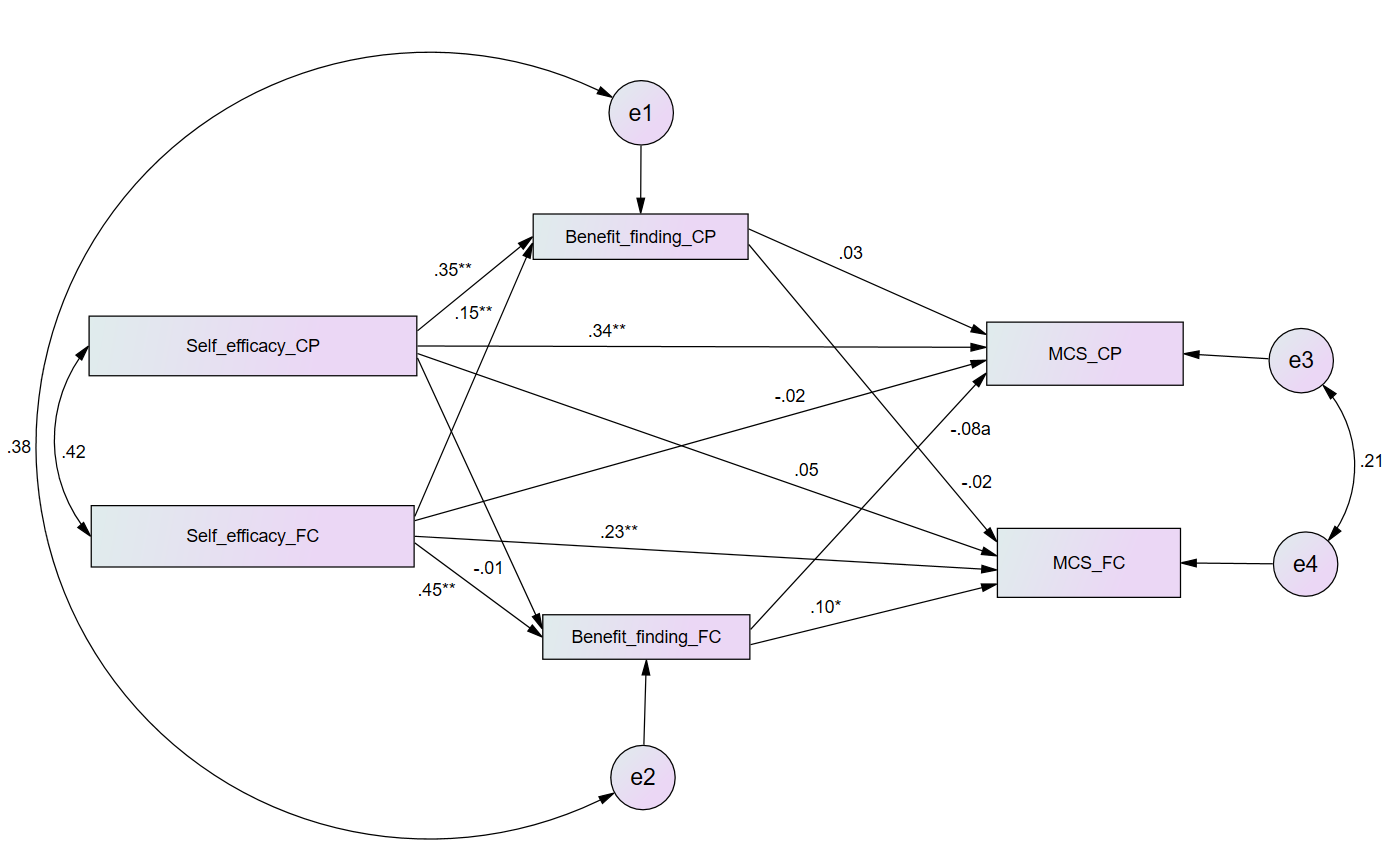


** P＜0.05; ** P＜0.01; a: P=0.072*

***P<0.01*

*Model a: submodel PCS*


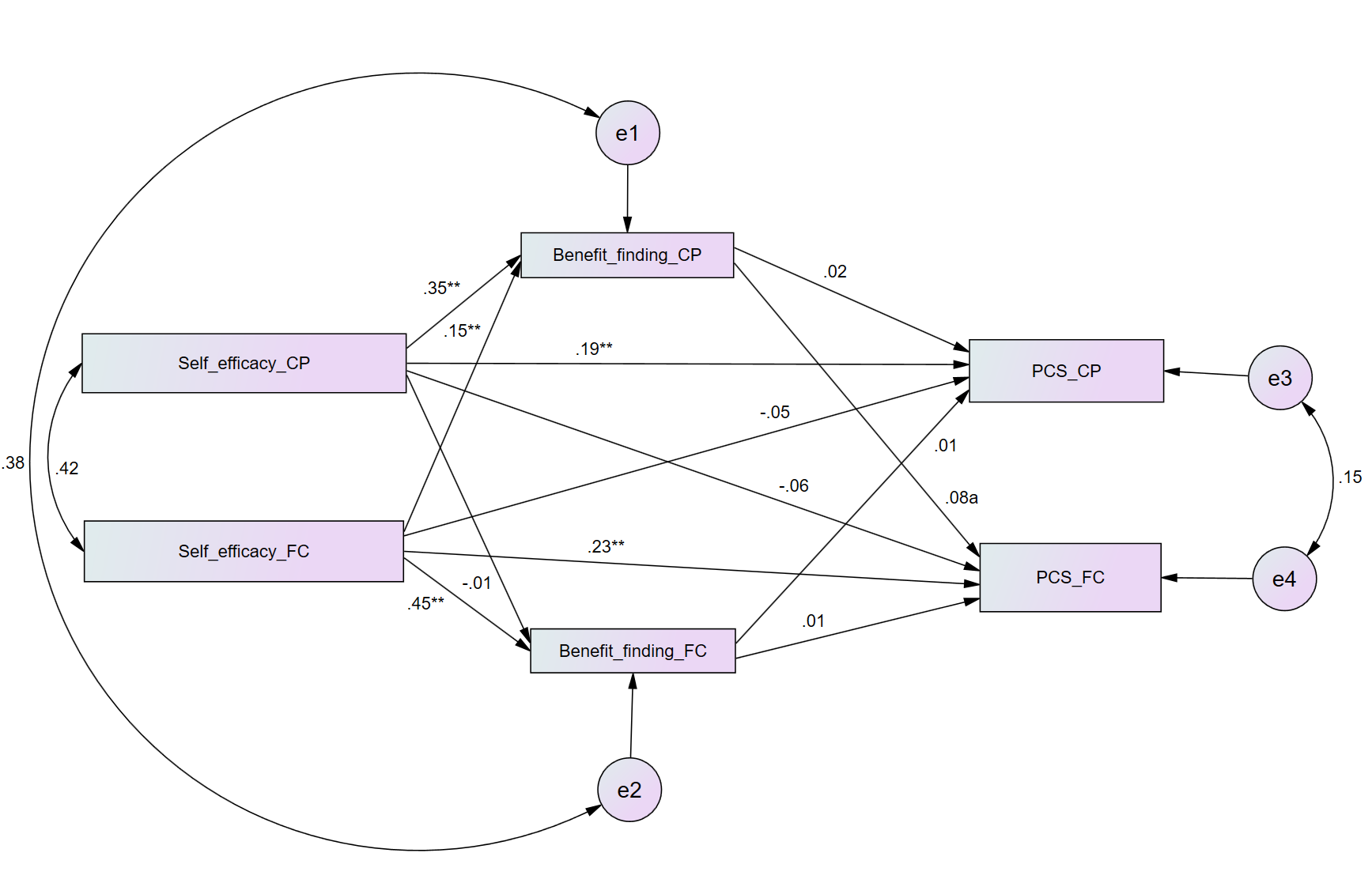


** P＜0.05; ** P＜0.01; a: P=0.066*

***P<0.01*

**Figure S4.** Two submodels for testing the impact of self-efficacy on Quality of life mediated by Psychological distress (Model b)

*Model b: submodel MCS*


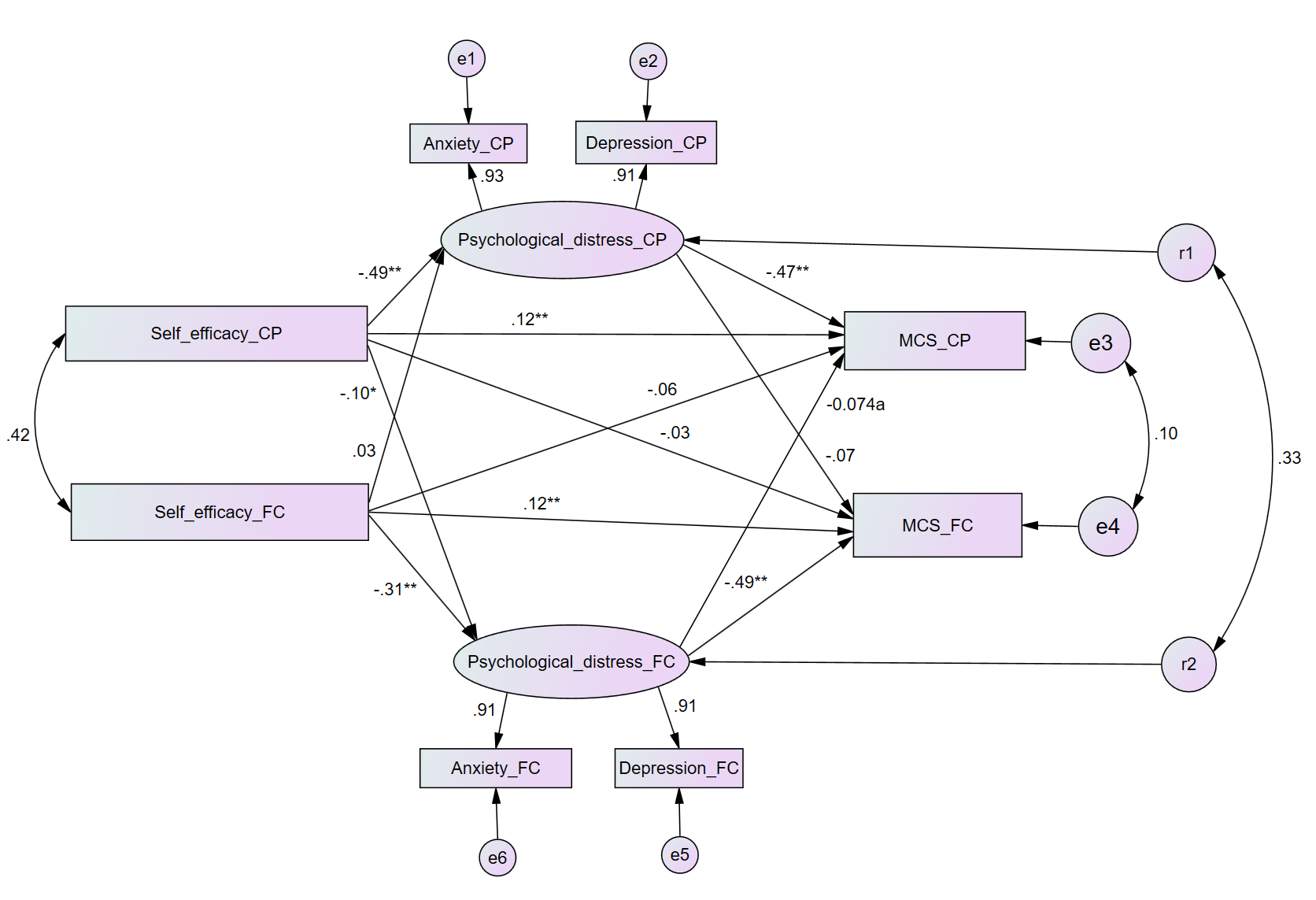


** P＜0.05; ** P＜0.01; a: P=0.069*

***P<0.01*

*Model b: submodel PCS*

** P＜0.05; ** P＜0.01*

***P<0.01*


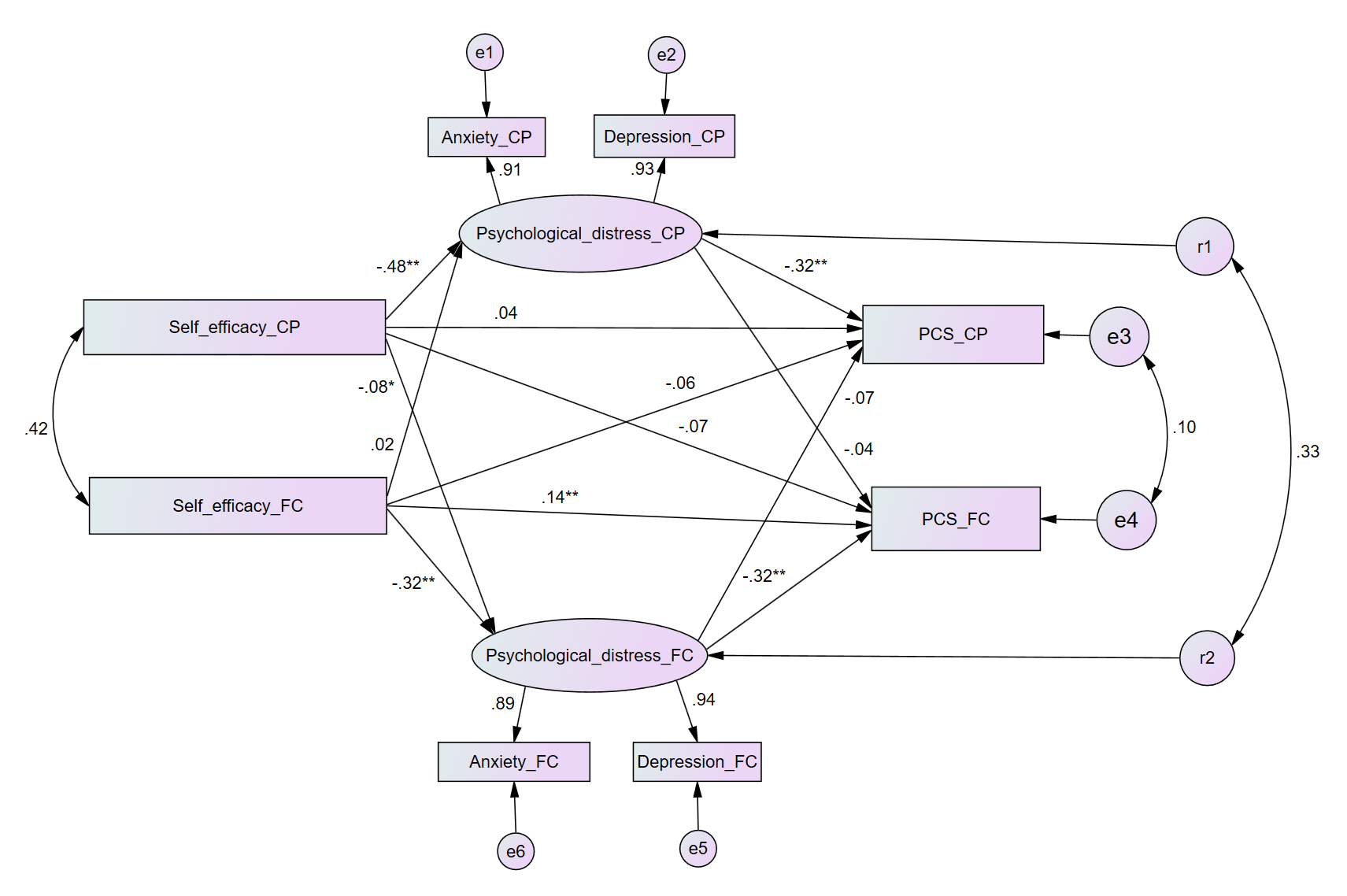

Supplement: Supplementary Materials — Figure S1: theoretical model in testing the impact of self-efficacy on Quality of life mediated by Benefit finding (Model a); Figure S2: theoretical model in testing the impact of self-efficacy on Quality of life mediated by Psychological distress (Model b); Figure S3: two submodels for testing the impact of self-efficacy on Quality of life mediated by Benefit finding (Model a); Figure S4: two submodels for testing the impact of self-efficacy on Quality of life mediated by Psychological distress (Model b). [file 3073358.f1.doc]
